# Modified liver mobilization for the treatment of renal cell carcinoma with thrombosis involving the intrahepatic inferior vena cava

**DOI:** 10.1186/1477-7819-12-131

**Published:** 2014-04-29

**Authors:** Zhijian Han, Changjun Yin, Xiaoxin Meng, Qiang Lu, Xiaobing Ju, Jie Li, Chao Qin, Pengfei Shao, Rijin Song, Pei Lu, Bianjiang Liu, Jiexiu Zhang, Min Gu

**Affiliations:** 1 Department of Urology, The First Affiliated Hospital of Nanjing Medical University, 300 Guangzhou Road, Nanjing 210029, China

**Keywords:** Renal cell carcinoma, Modified liver-mobilization technique, Thrombosis, Intrahepatic inferior vena cava, Father clamp

## Abstract

**Background:**

We aimed to evaluate the feasibility and clinical significance of using a modified liver-mobilization technique to treat renal cell carcinoma (RCC) combined with intrahepatic inferior vena cava (IVC) thrombosis.

**Methods:**

A total of 11 level III thrombus patients underwent radical nephrectomy with resection of the tumor thrombus from intrahepatic IVC. A father clamp was used in combination with hepatic portal blocking to control the IVC.

**Results:**

The intraoperative mortality and postoperative complications were reduced in 11 cases of RCC with intrahepatic IVC thrombosis. The mean blood loss was 800 mL, and mean patient hospital stay was 13 days. Follow-up was conducted for one to four months, with only two cases of recurrence recorded.

**Conclusions:**

The proposed modified liver-mobilization technique could safely and effectively treat RCC and reduce intrahepatic IVC thrombosis.

## Background

Inferior vena cava (IVC) involvement is present in 4 to 15% of patients with renal cancer carcinomas (RCC) [1]. The IVC involvement may be in the form of blood clots alone or as a combination of blood clots and tumor tissues. The invasion of the IVC wall occurs in 43 to 64% of tumor cases [2,3]. To date, the surgical removal of the kidney and the IVC thrombus are the only known methods to cure these tumors. A five-year survival rate of 30 to 70% can be achieved with such patients, in the absence of lymph node invasion or distant metastasis [4-6]. The level of IVC involvement has little effect on the survival rate of patients undergoing complete resection [7-9]. Our laboratory has been using liver transplant techniques to resect tumors and IVC thrombosis for the past 15 years. In the present study we used a modified liver-mobilization technique to treat 11 cases of RCC with intrahepatic IVC thrombosis, without opening the chest cavity or blocking the supradiaphragmatic IVC. The patients with RCC and IVC involvement were evaluated based on their clinical features, diagnostic modalities, surgical approaches, perioperative mortality, perioperative morbidity, and long-term outcomes.

## Methods

**Figure 1:**
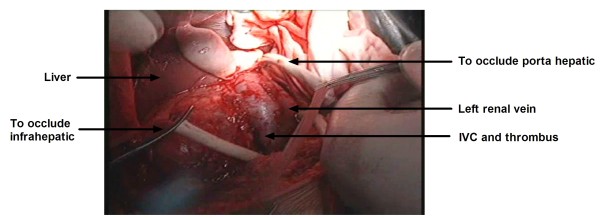
**The tourniquet loops are placed in the proper order: the infrarenal vena cava and the left renal vein are controlled.** IVC, Inferior vena cava.

**Figure 2:**
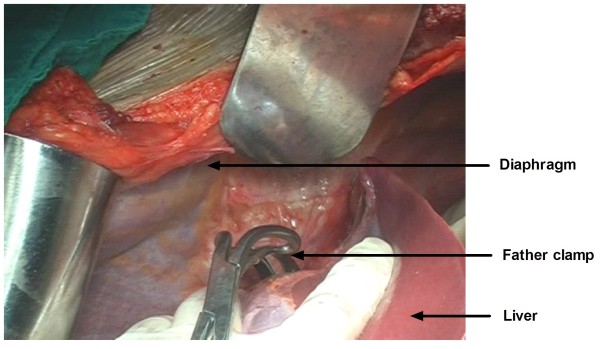
A father clamp is placed vertically across the Inferior vena cava.

The study was approved by the Institutional Review Board of the Nanjing Medical University, Nanjing, China. To treat the IVC thrombus, a subcostal incision of approximately the width of two fingers was made below the right costal margin and laterally extended to the midaxillary line. A framed self-retaining retractor was positioned by splaying it laterally toward the axillae to elevate the costal margins. After mobilizing the ascending colon, we ligated the involved renal artery over time. The arterial ligation consequently reduced the blood loss. Liver mobilization was started by dissecting the ligamentum teres, which were then divided. Traditionally, the falciform ligament is divided with cautery. This incision was made up to the right superior coronary ligament before it bypassed to the left side, thereby dividing the left triangular ligament. In the proposed modified and simplified liver-mobilization technique, we simply divided the falciform ligament to expose the whole suprahepatic IVC without having to incise the entire right superior coronary and left triangular ligaments. Dissection of the suprahepatic IVC is usually performed during liver transplantation to enable the use of a father clamp, which blocks the suprahepatic IVC. During liver mobilization, the right inferior coronary and hepatorenal ligaments are both incised to make the liver roll to the left, as described for liver transplantation. Minimally invasive procedures are achieved with this technique. Opening the lesser omentum would allow for the control of the porta hepatis with a tourniquet loop when necessary. This tourniquet loop temporarily occludes the blood inflow to the liver. Surgeons are advised to wait and allow the liver to decompress before applying other vascular clamps. The tourniquet loops were placed in the proper order. First, the infrarenal vena cava and the left renal vein were controlled (Figure 1), before a father clamp was placed vertically across the IVC (Figure 2). The IVC wall was incised upward from the opening of the renal vein to the third hepatic hilum. The tumor was then removed (mobile tumor thrombus) or dissected from the IVC wall (adherent tumor thrombus). After the removal of the tumor thrombus, the vena cava was closed with 4-0 polypropylene. Heparin saline was first injected into the opened IVC to wash out tumor tissue residues before closing the incision. Normal blood flow was thereafter reestablished in the liver.

To treat the left RCC, the right subcostal incision was extended below the left costal margin as far as necessary, as well as vertically extended in the midline to the xiphoid process. The exposure of the left kidney was started by mobilizing the descending and transverse colons from the spleen. The spleen and pancreas were then mobilized toward the midline. After ligation of the left renal artery, gonadal vein, and the ureter, the tumor was dissected from the Gerota’s fascia. The left kidney was freed and linked only by the occluded renal vein. The ascending colon and IVC were exposed. After the removal of the IVC thrombus, the entire left RCC was removed from the body.

## Results and discussion

A total of 11 level III thrombus patients underwent radical nephrectomy with resection of tumor thrombus from intrahepatic IVC. The mean age was 56 years (range: 44 to 75 years). The male-to-female ratio was 4.5:1, and the right-to-left ratio was also 4.5:1. Gross hematuria was the most common presenting symptom (six patients; 55%), followed by ipsilateral flank pain (three patients; 27%) and the presence of an abdominal mass (three patients; 27%). Only one patient presented with weight loss. The mean discharge of serum creatinine was 128 μmol/L (range: 76 to 197.5 μmol/L; normal: 44 to 136 μmol/L). Perioperative hemodialysis was not required by any of the patients. The level of thrombus on the computed tomography (CT) and magnetic resonance imaging (MRI) scans was well correlated with the intraoperative findings. The IVC tumor was completely extracted in all patients.

The mean operative time was 4 hours and 29 minutes (range: 2 hours and 50 minutes to 6 hours and 12 minutes), whereas the average time of liver ischemia was 15 minutes (range: 11 to 20 minutes). The average estimated blood loss (EBL) was 950 ± 876 ml (range: 550 to 3500 ml). The number of transfusions ranged from 3 to 18 U, with a mean of 5.2 ± 3.7 U.

All patients had an uneventful postoperative course, with no perioperative death or severe complications. Perioperative pulmonary embolisms did not occur, indicating that the IVC tumor was completely removed. The pathological outcome of the tumors from all patients was clear cell carcinoma, grade I to II. Lymph node metastasis was not observed in any of the patients. A mean follow-up of 22 months (range: 1 to 48 months) was achieved in nine patients (82%), with a median follow-up of 21 months. Among these patients, no evidence of disease was observed. Metastasis caused death in two patients, one after 15 months and another after 28 months. The overall three-year cancer-related mortality was 18%.

At least one of the three main symptoms of RCC (hematuria, flank pain, or abdominal mass) was apparent in most of the patients with combined RCC and intrahepatic IVC thrombosis. Simultaneously, symptoms of IVC occlusion, such as lower limb edema and varicocele (especially on the left side), accompanied the disease. Radiographic diagnoses were made by CT, MRI, or ultrasound techniques. The cardiac, renal, and respiratory status was evaluated before each operation. The thrombus was confirmed using MRI scans, which were helpful for assessing the involvement of the renal vein and IVC.

**Figure 3:**
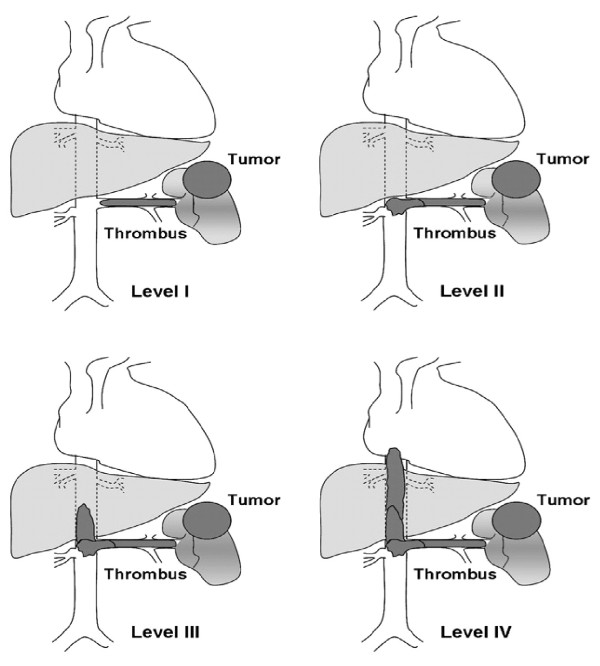
**The Mayo classification of macroscopic venous invasion in renal cell carcinoma **[10]**.** Level I: Tumor thrombus is either at the entry of the renal vein or within the IVC < 2 cm from the confluence of the renal vein and the IVC. Level II: Thrombus extends within the IVC >2 cm above the confluence of the renal vein and IVC but still remains below the hepatic veins. Level III: Thrombus involves the intrahepatic IVC. The size of the thrombus ranges from a narrow tail that extends into the IVC to one that fills the lumen and enlarges the IVC. Level IV: Thrombus extends above the diaphragm or into the right atrium. IVC, inferior vena cava.

Several classification systems have been used for vascular involvement in RCC. The Mayo classification (Figure 3) is a commonly used system that consists of four categories based on the extension of the thrombus [8]. Vascular involvement is classified as Level I when a thrombus is limited to the renal vein or less than 2 cm within the IVC. In Level II, the thrombus extends to more than 2 cm within the IVC, above the confluence of the renal vein and IVC, but remains below the hepatic veins. Level III is when the thrombus involves the intrahepatic IVC, whereas Level IV is when the thrombus extends above the diaphragm or into the right atrium. The selected surgical approach is based on the proper assessment of the venous extension.

Blancardus [10] was the first to describe IVC involvement of tumors. Only a few sporadic reports on the successful excision of a tumor thrombus in the IVC have been recorded;the operative mortality was too high, such that the technique was deemed as futile. In 1971, Skinner *et al.*[4] first described the thoracoabdominal approach, but this procedure caused the patients much pain. Cummings *et al.*[11] described a novel technique for IVC tumor thrombectomy in 1979; the vascular isolation of the IVC from the right atrium to the pelvis was achieved by temporary circulatory arrest of the lower torso. A neoplastic thrombus was excised from the intrapericardial IVC under direct vision. A midline abdominal incision was then made and extended from the cephalad into a sternotomy. However, this procedure caused severe trauma. In 1994, Marsh and Lange [12] applied liver transplant and organ procurement techniques to difficult upper abdominal urological cases. Ciancio et al. [13] described the piggyback technique in 2000, as well as the conventional technique of liver mobilization through a modified cruciate incision by employing the Rochard retractor; this technique was useful for gaining access to the retrohepatic IVC. Subsequently, a series of articles have been published to describe various conditions for dealing with renal tumors with an IVC thrombus [14-16].

In this study, we used a modified and simplified liver-mobilization technique to treat RCC with IVC thrombosis. We divided the falciform ligament to expose the entire suprahepatic IVC, without having to incise the right superior coronary ligament and the left triangular ligament. The suprahepatic IVC is usually dissected in liver transplantation in order to make room for a father clamp. Opening the lesser omentum allowed for a tourniquet loop to control the porta hepatis. A tourniquet loop was performed to temporarily occlude blood inflow to the liver and to allow for liver decompression. The veins were blocked in the proper order. The infrarenal vena cava and the normal renal vein were controlled before a father clamp was placed vertically across the IVC. The IVC was incised upward from the opening of the renal vein to the third hepatic hilum. The tumor was then removed (mobile tumor thrombus) or dissected from the IVC wall (adherent tumor thrombus). After the removal of the tumor thrombus, the vena cava was closed with 4–0 polypropylene.

This modified liver-mobilization technique avoids the need for a thoracoabdominal approach or sternotomy, and the surgical complications are reduced. For a right renal tumor, only a subcostal incision needs to be made at approximately the width of two fingers below the right costal margin, which is laterally extended to the midaxillary line. For left renal tumors, the right subcostal incision is made below the left costal margin, as far as is necessary, and further vertically extended in the midline to the xiphoid process. Before opening the IVC, 4–0 polypropylene is used to suture two ends of the incision. The incision includes the renal vein orifice, which allows for the convenient excision of the renal vein and the removal of the IVC thrombus *en bloc*. To avoid thrombus residue, a suction tube is inserted into the IVC cavity when removing the tumor. Heparin saline is repeatedly injected to wash out tumor residues completely before closing the IVC. Should the tumor invade the distal IVC, or even the common iliac veins, the distal veins may be resected and ligated.

## Conclusions

The resection of renal tumors with a Level III IVC thrombus has become feasible with the advancement of surgical techniques. The application of the modified liver-mobilization technique facilitates the effective exposure and proximal control of the IVC for removing a Level III thrombus. This technique greatly avoids complications and increases positive outcomes in tumor removal.

## Consent

Written informed consent was obtained from the patient for the publication of this report and any accompanying images.

## Abbreviations

IVC: inferior vena cava; RCC: renal cell carcinoma.

## Competing interests

The authors declare that they have no competing interests.

## Authors’ contributions

CY conceived the study, participated in the design and coordination of the study and drafted the manuscript. ZH, PL, BL contributed in the study design, literature search, data analysis, manuscript writing, editing and submission of the manuscript. CY, XM, QL, XJ, JL, CQ, PS, JZ, RS, MG participated in performed the procedure. CY, MG, supervised the study and contributed in data analysis, manuscript writing and editing. All the authors read and approved the final manuscript.
